# Comparative Fitness of a Parent *Leishmania donovani* Clinical Isolate and Its Experimentally Derived Paromomycin-Resistant Strain

**DOI:** 10.1371/journal.pone.0140139

**Published:** 2015-10-15

**Authors:** Sarah Hendrickx, Annelies Leemans, Annelies Mondelaers, Suman Rijal, Basudha Khanal, Jean-Claude Dujardin, Peter Delputte, Paul Cos, Louis Maes

**Affiliations:** 1 Laboratory for Microbiology, Parasitology and Hygiene (LMPH), University of Antwerp, Antwerp, Belgium; 2 BP Koirala Institute of Health Sciences, Dharan, Nepal; 3 Molecular Parasitology Unit, Institute of Tropical Medicine Antwerp, Antwerp, Belgium; 4 Department of Biomedical Sciences, University of Antwerp, Antwerp, Belgium; Royal Tropical Institute, NETHERLANDS

## Abstract

Paromomycin has recently been introduced for the treatment of visceral leishmaniasis and emergence of drug resistance can only be appropriately judged upon its long term routine use in the field. Understanding alterations in parasite behavior linked to paromomycin-resistance may be essential to assess the propensity for emergence and spread of resistant strains. A standardized and integrated laboratory approach was adopted to define and assess parasite fitness of both promastigotes and amastigotes using an experimentally induced paromomycin-resistant *Leishmania donovani* strain and its paromomycin-susceptible parent wild-type clinical isolate. Primary focus was placed on parasite growth and virulence, two major components of parasite fitness. The combination of *in vitro* and *in vivo* approaches enabled detailed comparison of wild-type and resistant strains for which no differences could be demonstrated with regard to promastigote growth, metacyclogenesis, *in vitro* infectivity, multiplication in primary peritoneal mouse macrophages and infectivity for Balb/c mice upon infection with 2 x 10^7^ metacyclic promastigotes. Monitoring of *in vitro* intracellular amastigote multiplication revealed a consistent decrease in parasite burden over time for both wild-type and resistant parasites, an observation that was subsequently also confirmed in a larger set of *L*. *donovani* clinical isolates. Though the impact of these findings should be further explored, the study results suggest that the epidemiological implications of acquired paromomycin-resistance may remain minimal other than the loss of one of the last remaining drugs effective against visceral leishmaniasis.

## Introduction

In the last decade, management of visceral leishmaniasis (VL) in the Indian subcontinent became severely compromised by the mounting level of antimony (Sb^V^) treatment failure [1]. To combat this widespread resistance and reduce the number of VL infections, the Kala-azar elimination program was launched in India, Nepal and Bangladesh in 2005 [2]. In this program, miltefosine (MIL) was listed as first-line alternative for Sb^V^-treatment, whereas paromomycin (PMM), which was licensed for VL in 2006, is now considered an attractive alternative for Sb^V^ in combination therapy because of its well-defined efficacy and safety profile [3,4]. Although its use is yet still restricted, the lack of new drugs in the development pipeline will certainly lead to a more regular application of PMM in the future. However, its efficacy should be closely monitored as resistance may emerge once it will become more routinely used in the field. Extensive exploratory work regarding PMM-resistance has already been carried out in frame of the European Kaladrug-R project that aimed at identifying novel methods to monitor and evaluate drug resistance in the field [5–7]. In depth study of PMM-resistance will contribute to manage its emergence or even reveal potential targets that might lead to the design of novel drugs [8]. Linked to its recent introduction onto the market, PMM-resistant clinical isolates have not yet been isolated, highlighting the need for proactive laboratory studies on PMM-resistance. These should enable the characterization of underlying molecular resistance mechanisms in combination with a better understanding of the phenotypic behavior of R parasites towards emergence and spreading potential [9]. Most current research generally aims to identify genomic resistance markers as molecular resistance surveillance tools, but marker identification and validation has proven to be a long and difficult process [10,11], among others because of the complex and multifactorial nature of the phenomenon [12]. Complementary to this, resistance can also be approached phenotypically where the influence of resistance on apparent parasite fitness parameters like growth and infectivity are explored. The occurrence of resistant parasites with characteristics for enhanced spread and virulence has already been postulated for Sb^V^-resistant [13–19] and MIL-resistant *L*. *donovani* strains [20]. Although fitness is actually a complex interplay of many different factors influencing survival, reproduction and transmission between hosts in a given environment [21,22], *Leishmania* parasite fitness is especially influenced by its ability to reproduce (*e*.*g*. growth characteristics) and spread (*e*.*g*. infectivity) to other hosts [9] and has mainly been studied on (easy cultivable) promastigotes that must be considered as much less relevant compared to the intracellular amastigote stage [9]. Furthermore, *in vitro* culture conditions such as nutrient availability, pH of the medium and culture age have a large and often underestimated impact on phenotypic promastigote behavior [23,24]. It is essential to focus on the intracellular amastigote stage whenever possible [25]. When dealing with clinical isolates of which generally only promastigotes are available, more standardization in study design must be considered [26].

**Fig 1:**
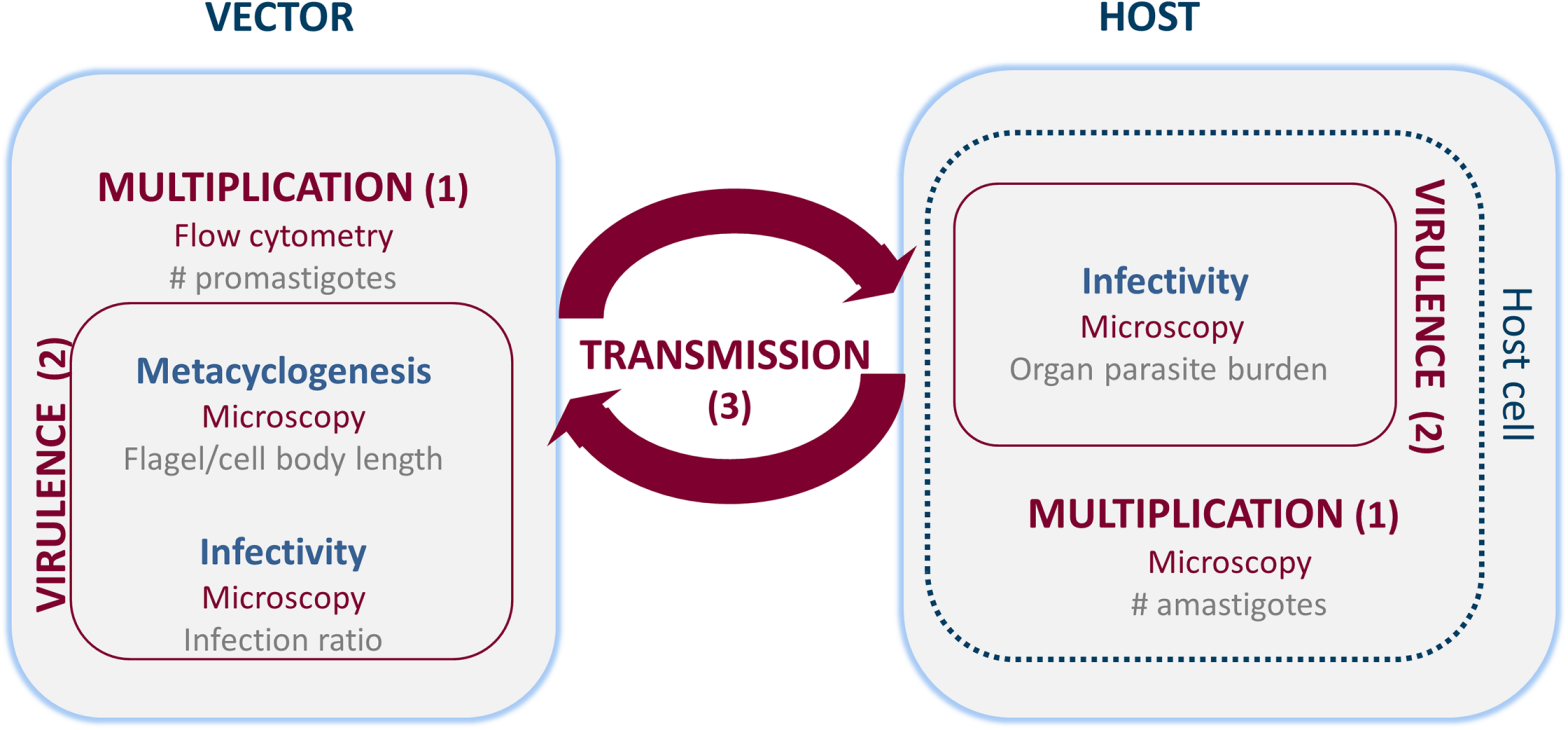
Overview of the different parameters studied for the evaluation of parasite fitness. Parasite fitness depends on three main factors: (1) growth or multiplication, (2) virulence and (3) transmission. In order to assess these factors, parasite growth, metacyclogenesis and infectivity were explored using *in vitro* and *in vivo* laboratory methods.

The present laboratory study specifically aimed to establish a refined methodology to evaluate parasite fitness on both (extracellular) promastigote and intracellular amastigote level (Fig 1) using 1/ flow-cytometry and microscopy to assess parasite growth/multiplication and 2/ metacyclogenesis and *in vitro* and *in vivo* infectivity to characterize virulence. This complementary set of assays was then applied for direct comparison of fitness of an experimentally induced PMM-resistant (R) with its PMM-susceptible parent wild-type strain (WT) [5]. For comparative evaluation of *in vitro* infectivity, a larger set of *L*. *donovani* strains was included (*see supplementary material*).

## Materials and Methods

### Ethical statement

The use of laboratory rodents was carried out in strict accordance to all mandatory guidelines (EU directives, including the Revised Directive 2010/63/EU on the Protection of Animals used for Scientific Purposes that came into force on 01/01/2013, and the declaration of Helsinki in its latest version) and was approved by the ethical committee of the University of Antwerp, Belgium (UA-ECD 2010–17 (18-8-2010).

### Parasite strains

Since PMM has been assigned as replacement therapy in patients who no longer respond to Sb^V^, an isolate with Sb-resistant background was selected for experimental PMM-resistance selection [5] and the subsequent fitness assays. The parent strain (MHOM/NP/03/BPK275/0) was isolated from the bone-marrow of a Nepalese patient upon Sb^V^-treatment failure, cloned and typed as *L*. *donovani* based on CPB-PCR RFLP [27]. Promastigotes were routinely cultured in T25 culture flasks containing 5 ml of HOMEM medium (Invitrogen, UK) supplemented with 10% heat inactivated fetal calf serum (iFCS). The same batch of medium was used for all experiments. A selection of additional *L*. *donovani* strains was included to comparatively check the *in vitro* intracellular growth characteristics (*see supplementary material*).

### Animals

Female Balb/c mice (BW 20–25 g) were purchased from Janvier (France) and kept in quarantine for at least 5 days before infection. Food for laboratory rodents and drinking water were available ad libitum. The animals were randomly allocated to 2 experimental units of 6 animals each.

### Resistance induction

The induction of PMM-resistance at intracellular amastigote level has previously been described [5]. The induced resistance was stable both after long-term cultivation of the promastigotes *in vitro* and after two successive *in vivo* passages in the hamster. After resistance induction, the resulting population was cloned and the most resistant clone (cl-1) was selected for the parasite fitness studies.

### Flow cytometric assessment of promastigote growth

Growth curves were constructed to compare the *in vitro* growth profile of resistant (R) and susceptible wild-type (WT) promastigotes. Clustering promastigotes were separated by repeated needle passage (21G x 1½”, 0.8 x 40mm, 25G x 5/8”, 0.5 x 16mm) and diluted in phosphate buffered saline (PBS) for flow cytometric (FCM) counting, using a FACSCalibur® flow cytometer (BD Biosciences, NJ, USA) with addition of CountBright absolute counting beads (CB; Molecular Probes®, OR, USA) as internal standard for quantification of the exact volume analyzed. All FCM samples were measured *in duplo* and further analyzed using the BD CellquestPro® software. To establish the growth curve, promastigotes were inoculated in 5 ml HOMEM at exact 5x10^5^ promastigotes/ml with subsequent quantification of three biological replicates every 24h. The average promastigote density for each time point was used to draw the growth curves.

### Morphological assessment of metacyclogenesis

Promastigote metacyclogenesis is pivotal for adequate infectivity i*n vitro* and *in vivo* [28]. The overall progression of this process is accompanied by typical morphological changes of the promastigote cell body whereby promastigotes are considered metacyclic when the flagella/cell body length ratio exceeds 2 [13]. Every 24h, a drop of promastigote suspension was put on a glass coverslip, fixed with methanol and stained with Giemsa. Promastigotes were visualized *ad random* with bright field microscopy (Axiovert 200m®, Carl Zeiss) using the Zeiss Axiocam MRm®. The cell body and flagella length of at least 50 promastigotes per sample were measured using the Axiovision® software.

### Microscopic evaluation of *in vitro* and *in vivo* infectivity

The *in vitro* infectivity of R and WT metacyclics was comparatively evaluated by determination of their maximal infection potential for macrophages [29]. Promastigotes at different phases in their growth curve were counted by FCM and used to infect primary peritoneal mouse macrophages, adopting a 15/1 parasite/macrophage ratio. To correct for the variable number of dead promastigotes, live/dead staining was carried out using the single-stain viability dye TO-PRO®-3 iodide (Molecular probes®, OR, USA) [30]. Promastigote uptake was enhanced by reducing the culture volume to 30 μl and macrophages were incubated at 37°C in 5% CO_2_ for 4 hours, after which 100 μl of macrophage medium was added to each well. The macrophages were fixed 24h post-infection with methanol and stained with Giemsa. At least 100 macrophages were evaluated to determine the average number of intracellular amastigotes per macrophage and the percentage of infected macrophages.

To evaluate *in vivo* infectivity of promastigotes, for each strain 6 female Balb/c mice were infected intracardially with 2 x 10^7^ metacyclics. Their general health condition and body weight were monitored twice weekly although the infection with *L*. *donovani* remains subclinical. At the peak of infection, estimated at twenty-eight days post-infection, all animals were euthanized with a CO_2_ overdose and amastigote burdens in the target organs liver and spleen were determined to compare peak organ burdens upon infection with R and WT parasites. The organs of individual animals were weighed and impression smears were stained with Giemsa for microscopic enumeration of the average number of amastigotes per cell by counting a minimum of 500 nuclei. The results are expressed as Leishman-Donovan Units (LDU) [31]. The viability of the amastigotes was qualitatively assessed using the promastigote back transformation assay by placing a small piece of spleen and liver tissue in HOMEM promastigote medium at room temperature for up to 2 weeks.

### Microscopic assessment of intracellular amastigote multiplication

Primary peritoneal mouse macrophages were collected from female Swiss mice, seeded in 96-well plates with 30,000 macrophages/well in 100 μl of RPMI-1640 (Invitrogen, UK) [32] and infected 24 hours later with metacyclic promastigotes based on the criteria and quantified by FCM as described above. Amastigote growth was evaluated by staining infected macrophages every 24h with Giemsa and determining the infection index according to the formula:

$$\text{Infection index}=\frac{\text{\# amastigotes counted}}{\text{total \# macrophages counted}}$$ (1)

To correct for differences in baseline infectivity, the infectivity 24h post-infection was used as an internal baseline control (T_0_). Amastigote multiplication ratios were calculated using the formula:

$$\text{Amastigote multiplication ratio}=\frac{\text{infection ratio at}\;{\text{T}}_{\text{x}}}{\text{infection ratio at}\;{\text{T}}_{0}}$$ (2)

### Statistical analysis

All statistical analyses were performed using Graphpad Prism version 4.00 software. Statistical differences between WT and R parasites and between the different time points within one group were determined using 2-way ANOVA with Bonferroni post-hoc comparisons for parasite growth, parasite morphology and infection indices. Morphological and infection indices intergroup comparison was done using non-parametric Friedman test followed by Dunn's post-hoc comparisons. Tests were considered statistically significant if p<0.05 (*).

## Results

### Flow cytometric assessment of promastigote growth

**Fig 2:**
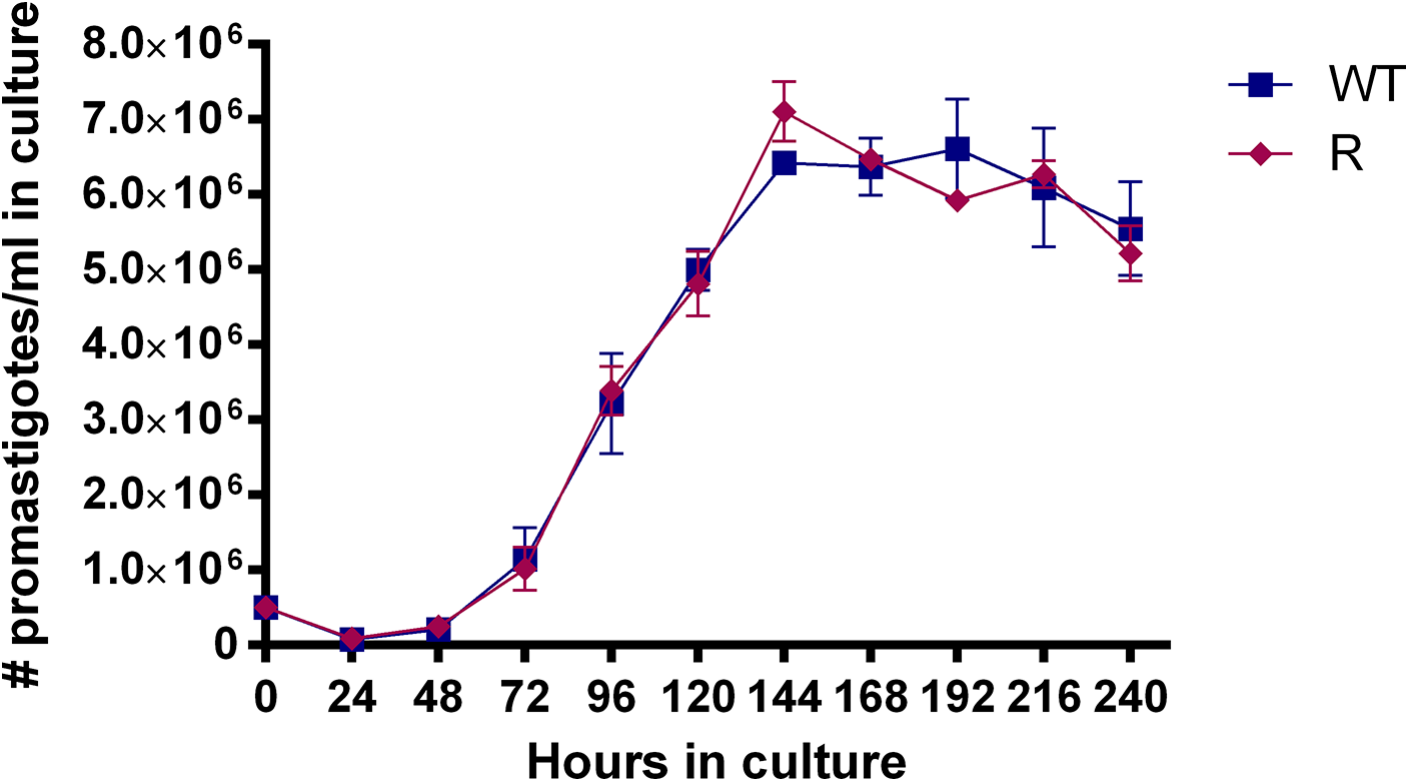
Promastigote growth curves of the paromomycin-susceptible wild type (WT) and its derived paromomycin-resistant (R) *Leishmania donovani* strain. Promastigotes reach stationary phase after 144h of cultivation. No growth differences are observed between WT and R (p<0.05). Results are expressed as the average ± the standard error of mean from three different experiments run in duplicate.

The average promastigote density in culture was determined every 24h by FCM. Both WT and R parasites behaved similarly in terms of *in vitro* multiplication (Fig 2). Under the stated culture conditions, promastigotes entered stationary phase after about 144h of cultivation.

### Morphological assessment of metacyclogenesis

**Fig 3:**
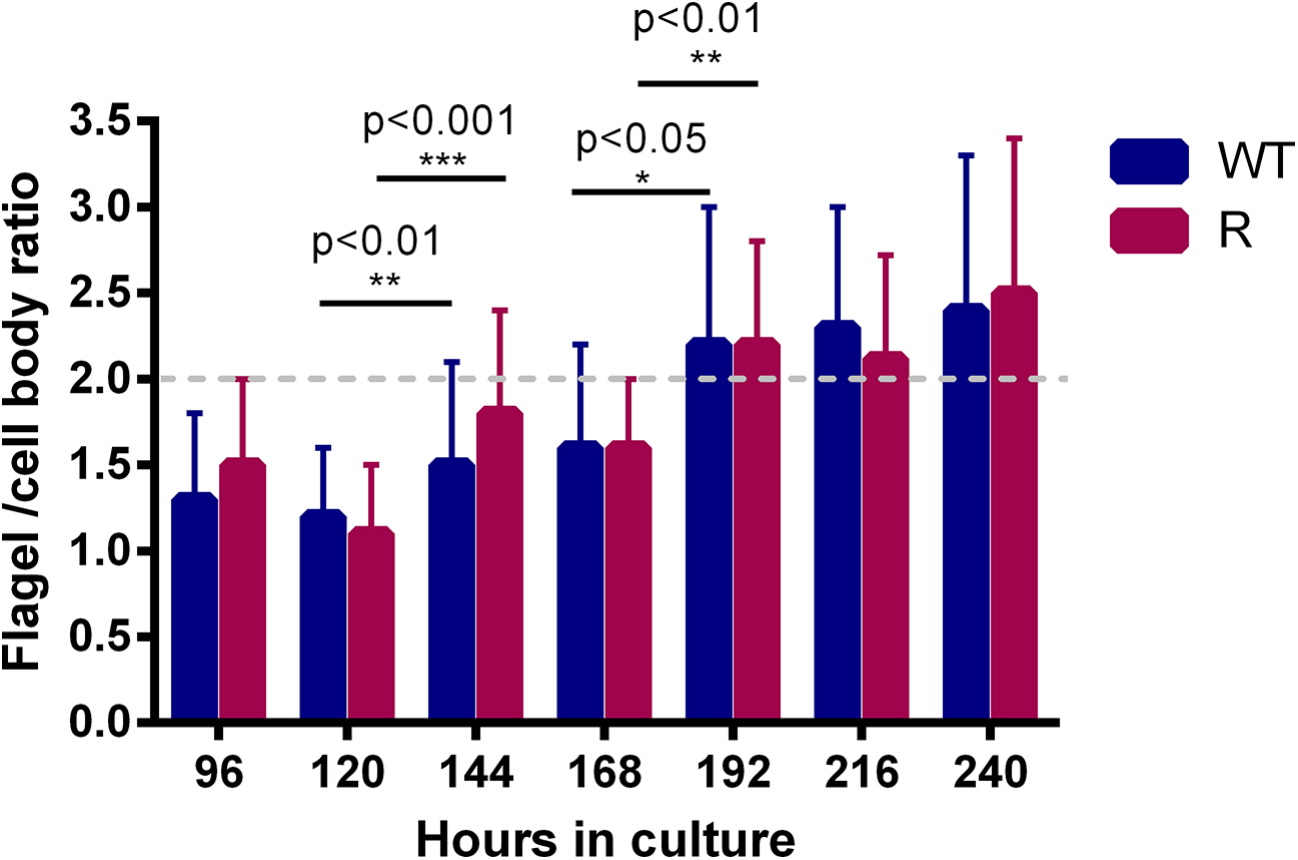
Metacyclogenesis of promastigotes. Based on morphological evaluation, promastigote are metacyclic after 192h in HOMEM culture. Differences between 120h and 144h and between 168h and 192h are statistically significant (p<0.05) both for WT and R parasites. No significant differences in morphology are demonstrated between WT and R parasites. Results are expressed as the average ± the standard error of mean from three different experiments run in duplicate.

Every 24h of cultivation, flagellum and cell body of at least 50 promastigotes were measured for determination of the flagellum/cell body ratio and promastigotes were considered fully metacyclic if the ratio was >2. Based on the latter, metacyclogenesis started after 144h and reached a maximum at 192h with about 80% of the promastigotes reaching the pre-set metacyclogenesis cut-off (Fig 3). Flagellum/cell body ratios were significantly different between 120h and 144h and between 168h and 192h of culture for both WT and R parasites. No significant differences in morphology could be demonstrated between WT and R parasites.

### Microscopic evaluation of *in vitro* and *in vivo* infectivity

**Fig 4:**
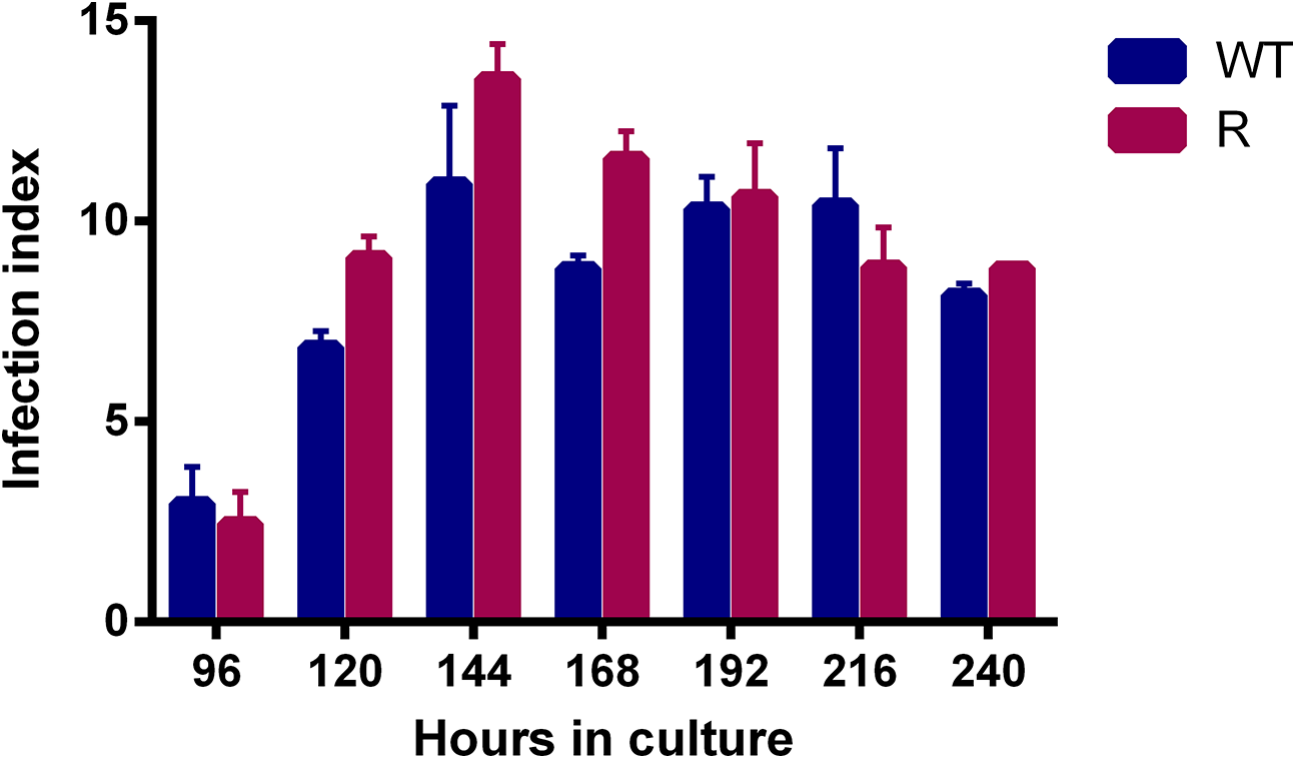
Infectivity of WT and paromomycin-R promastigotes for primary peritoneal mouse macrophages. **I**nfection indices are highest after 144h of cultivation for both strains,. There is no significant difference between WT and R for infectivity at the different time points (p>0.05). Results are expressed as the average ± the standard error of mean from three different experiments run in duplicate.

**Fig 5:**
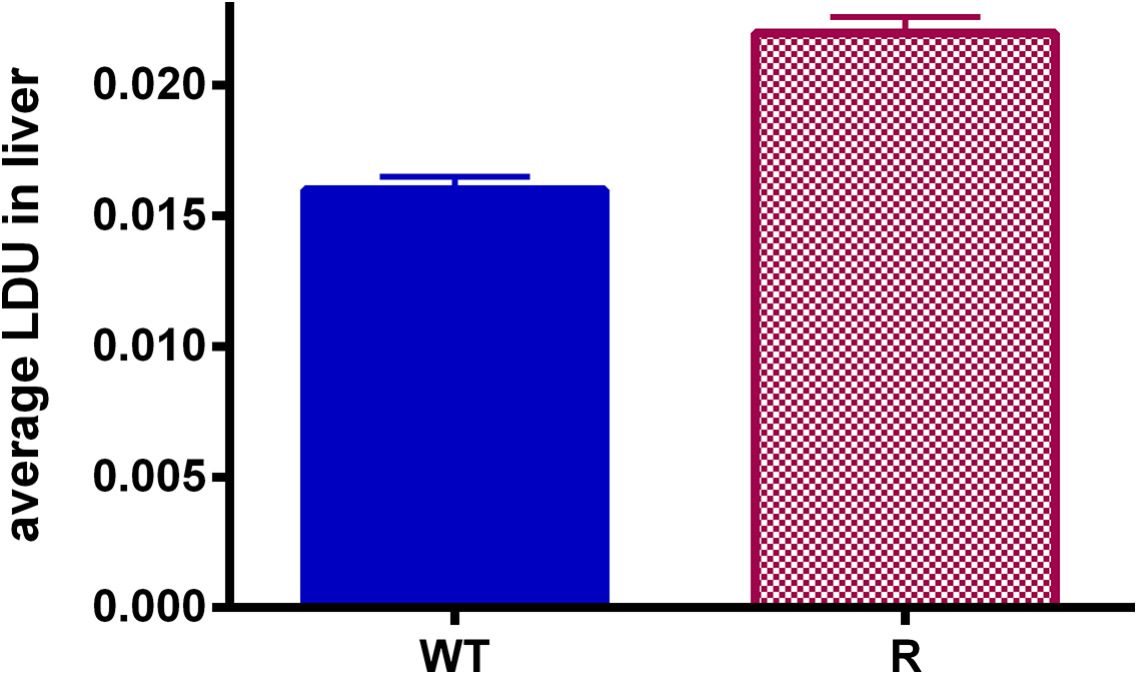
The average LDU in the liver of infected Balb/c mice at 28dpi. A significant difference was detected between WT (# = 6) and R (# = 6) parasite infection rates *in vivo* (p<0.001). However, overall infection levels were too low to draw well-founded conclusions on this observation. Infection levels were the result of three independent repeats and are expressed as the average ± the standard error of mean.

When comparing promastigote infectivity after various cultivation periods (Fig 4), *in vitro* infectivity indices reached a maximum after 144h of cultivation, hence coinciding with the peak of metacyclogenesis. For all evaluated time points, there is no significant difference in infectivity of WT and R parasites for primary peritoneal mouse macrophages. All infected Balb/c mice remained without symptoms and *in vivo* parasite burdens at 28 dpi were very low (Fig 5) since only few amastigotes were visible in the Giemsa-stained spleen and liver smears. Although the observed differences between WT and R parasites were statistically relevant, no firm conclusions can be drawn regarding *in vivo* virulence. The promastigote transformation assays of the liver and spleen were positive for all infected mice within 7 days after autopsy.

### Microscopic assessment of intracellular amastigote growth

**Fig 6:**
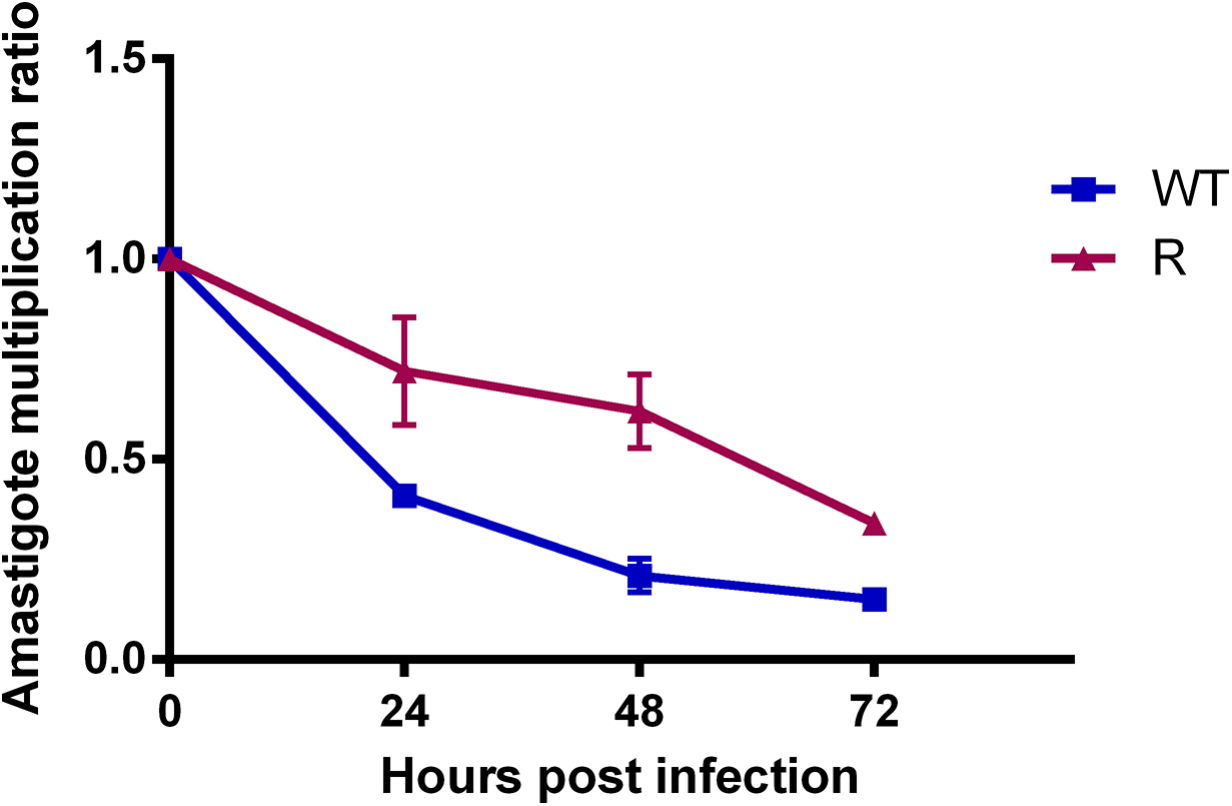
Amastigote growth of R and WT parasites in primary mouse macrophages after infection with optimal metacyclic promastigotes. A consistent decline in amastigote burden is observed in time with a statistical difference between WT and PMM-R parasites (p <0.05). Results are expressed as the average ± the standard error of mean from three different experiments run in duplicate.

After infection with optimal metacyclic promastigotes, the amastigote multiplication ratio was calculated at 24h intervals (Fig 6). A rather consistent decline in initial intracellular parasite burden was observed over time. To corroborate this observation, this assay was repeated for a broader selection of *L*. *donovani* strains of various origin and with different drug susceptibility profiles (Table A in S1 File). In addition to infection with metacyclic promastigotes, intracellular replication upon infection with *ex vivo* spleen-derived amastigotes (when available) was evaluated as well. Upon infection with these *ex vivo* amastigotes, a consistent rise in intracellular parasite burden was observed (Figure A in S1 File).

## Discussion

For over 60 years, Sb^V^-treatment remained the first-line therapy for VL despite its association with major drawbacks, such as parenteral treatment and severe adverse effects [33]. In addition, the therapeutic value of Sb^V^ has been jeopardized in endemic areas in India due to increasing treatment failure rates [34], which actually led to the recommendation to switch to other drugs such as amphotericin B deoxycholate, liposomal amphotericin B, miltefosine (MIL) and paromomycin (PMM) [2]. As current treatment options are limited and no new drugs may reach the market in the near future, existing therapeutics should be safeguarded with particular attention for drug resistance avoidance. In 2006, the aminoglycoside antibiotic PMM was licensed in India as a safe and affordable option for mono- and combination therapy of VL [3,4,35]. Within the knowledge that resistance to aminoglycoside antibiotics can easily be acquired in bacteria [36], there is ample reason to believe that emergence of PMM-resistance in *Leishmania* should be monitored with vigilance. After all, recent laboratory results have indicated that even in drug combinations, PMM-resistance can be fairly easily induced *in vitro* both in amastigotes and promastigotes [5,37,38]. Although consistent PMM-resistance in clinical isolates has not yet been reported probably because of the currently low drug pressure, the increasing use of PMM will undeniably trigger selection for resistance in the field.

To tackle this problem proactively, experimentally selected PMM-resistant strains can be used to unravel the potential impact on disease transmission and epidemiology. For example, genetic modifications in resistant parasites can be explored to discover mutations responsible for resistance, similar to the numerous studies that focused on genetic markers to monitor the spread and emergence of Sb^V^-resistant parasites [12,39–41]. On the other hand, it remains very difficult to validate such markers particularly since resistance is complex and often multifactorial. For the latter reasons, drug resistance is also investigated phenotypically preferably by comparing parasite behavior of susceptible wild-type (WT) and resistant (R) matched pairs. Increasing attention is currently being given to parasite fitness, which is an estimation of the parasite’s ability to reproduce and successfully transmit the disease. Development of drug resistance may indeed impact on parasite fitness by causing a competitive cost or benefit to the organism [42]. Generally, drug-resistant organisms tend to be less infective, less virulent or display a decreased transmission potential. In some organisms, *e*.*g*. in *Mycobacterium tuberculosis*, fitness is less affected [21]. More exceptionally, resistance may confer increased parasite fitness as has been demonstrated for diverse set (no pair-matched) Sb^V^-resistant *L*. *donovani* isolates [13–19]. Remarkably, the latter finding apparently does not apply to other antileishmania drugs or *Leishmania* species. For example, tunicamycin-resistant *L*. *mexicana* virulence did not change in comparison to sensitive parasites [43,44] and amphotericin B-resistant *L*. *mexicana* and glucantime-resistant *L*. *guyanensis* were clearly associated with a decrease in infectivity [45,46]. However, it must be noted that comparing relative fitness between different *Leishmania* strains is complicated and to a certain extent inappropriate since each strain has its own multifaceted characteristics and culture preferences. To guarantee a more accurate and valid comparison between strains, a well-standardized methodology is pivotal as was adopted in the present study where methodologies were used to standardize metacyclogenesis, parasite multiplication and infectivity using a matched pair of a PMM-susceptible parent WT strain and its derived PMM-resistant strain induced on intracellular amastigotes [5]. Using a same strain before and after resistance induction assures minimal genetic heterogeneity between both. Various additional measures were taken to rule out potential confounding parameters that are non-related to parasite fitness. For example, the same batch of medium was used for all experiments as it is well known that growth and differentiation can easily be influenced by the composition of the culture medium (*e*.*g*. pH and available nutrients) [23] and hence has an indirect but significant impact on metacyclogenesis, infectivity and ensuing virulence. Since long-term *in vitro* maintenance is known to decrease parasite virulence [34], the number of passages was kept as low (<20) as possible for both strains to ensure a comparability throughout the course of experiments. Because infectivity is strongly stage-dependent, the metacyclogenesis process of both strains was accurately monitored, which included flagellum/cell body measurements and assessment of host cell infectivity of stationary-phase promastigotes, taking viability into account by using flow cytometric live/dead quantification after TO-PRO^®^-3 iodide staining [30]. This way, initial infection ratios are the result of infections with equal numbers of viable metacyclics, a correction that is rarely adopted in comparable infectivity studies in literature.

By implementing the above methodologies and criteria, no differences between R and WT promastigotes could be revealed. Promastigote growth curves illustrate that both WT and R parasites display an identical growth pattern and start to enter stationary phase and metacyclogenesis after about 144h (Fig 2). In addition, WT and R promastigotes show no difference in *in vitro* infectivity, resulting in fully comparable intracellular amastigote burdens (Fig 4). Although promastigote morphology changed significantly between 120h and 144h, the pre-set cut-off value (>2) for metacyclogenesis was only reached after the second significant transformation between 168h and 192h. Of course, the pre-determined cut-off value is only an approximate number based on existing literature [8] in addition to the fact that morphological changes may most likely also be species-, population- and environment dependent. A more remarkable observation was the consistent decrease in the intracellular amastigote burden over time (Fig 6) which could also be observed in other *L*. *donovani* strains (*see supplementary material*), highlighting the obvious contrast between infection with metacyclic promastigotes or *ex vivo* amastigotes.

Despite different attempts to optimize/stimulate amastigote growth *in vitro*, such as more frequent renewal of the culture medium, no rise in parasite burden could be achieved. Accordingly, also the *in vivo* infectivity resulted in quite low LDU’s (Fig 5). Although metacyclogenesis was fully optimized within the context of the *in vitro* experiments, no satisfactory levels of infection could be obtained in Balb/c mice. Although the passage number of the *in vitro* cultures was kept as low as possible, intrinsic adaptation of the promastigotes to *in vitro* cultivation cannot be ruled out. Literature repeatedly mentioned loss of parasite virulence upon long- and even short-term cultivation *in vitro* [47,48]. Similarly, laboratory strains that have been maintained *in vivo* for a long time also exhibit difficulties to adapt to *in vitro* growth as promastigote This may explain why infection of animals with spleen-derived amastigotes is generally more successful compared to the use of *in vitro* grown metacyclic promastigotes. In the present study, it was unfortunately not possible to use spleen-derived amastigotes from infected donor animals because of the too low organ amastigote burdens (Fig 5).

Various research papers already documented that *in vitro* intracellular amastigote burdens tend to decline in time rather than to increase, a phenomenon that is generally overlooked in short-term assays [49–51]. However for drug screening purposes, the need for a dividing population has clearly been pointed out [52].

Based on our *in vitro* and *in vivo* results with the PMM-susceptible and PMM-resistant matched strains, no impact of resistance on parasite fitness could be demonstrated. These results are further supported by the fact that after *in vivo* passage of the originally induced polyclonal population, amastigotes with both R and S-phenotypes could be harvested (data not shown), endorsing that none of both phenotypes has overgrown the other. It is obvious that the present observations in one matched pair of WT and R *L*. *donovani* required further validation on a larger sample set, including Sb^V^-susceptible *L*. *donovani*, since Sb-resistance may facilitate the ease of resistance development by modulation of the cell membrane and thus potentially influence fitness outcome [53,54]. Preliminary results obtained upon comparison of a PMM-susceptible clinical isolate with Sb-susceptible background and its experimentally derived PMM-resistant isolate indicate no influence of the Sb-susceptibility background (data not shown).

Our results differ from those where fitness was evaluated in strains where PMM-resistance was induced on promastigotes. Resistant parasites revealed increased fitness compared to WT and was reflected by enhanced membrane fluidity, decreased drug accumulation and increased drug efflux by up-regulation of transporters related to drug resistance, such as MDR1 and MRPA and finally an increased stimulation of host IL-10 levels [7]. Although this study deals with a genetically different strain, these results obtained in amastigote- and promastigote-induced resistant strains once again emphasize the pivotal importance of the selection method. Noting that PMM-resistance could only be expressed at amastigote level leaving promastigotes fully susceptible upon R-selection on intracellular amastigotes [5] strongly motivates for using amastigote-based models whenever possible. Although laboratory-induced resistance can provide valuable insights into resistance mechanisms and potential consequences of resistance, conclusions based on laboratory-resistance should nevertheless still be interpreted with great caution until validation on large sets of field isolates becomes possible. Although more complex and requiring specialized research facilities, future fitness studies should also consider including the comparative assessment of the survival capacity and metacyclogenesis of drug-susceptible and -resistant parasites in the sandfly vector.

## Supporting Information

S1 File Evaluation of intracellular amastigote replication for a selection of *L*. *donovani* field isolates. Drug-susceptibility profile of the selected *L*. *donovani* field isolates to evaluate intracellular amastigote replication **(Table A).** Intracellular growth curves of the *L*. *donovani* field isolates and the reference lab strain. Using metacyclic promastigotes for infection, a decline in initial (24h) intracellular amastigote burden was observed for all the *L*. *donovani* strains tested. When *ex vivo* amastigotes (*) were used to infect host cells, a consistent increase in parasite burden was observed **(Fig A)**.
(DOCX)
