# Supplementary material for: Comparative Fitness of a Parent Leishmania donovani Clinical Isolate and Its Experimentally Derived Paromomycin-Resistant Strain
Source: PLoS One. 2015 Oct 15;10(10):e0140139. doi: 10.1371/journal.pone.0140139 (PMC4607421; doi:10.1371/journal.pone.0140139)
Supplement: S1 File — Drug-susceptibility profile of the selected L. donovani field isolates to evaluate intracellular amastigote replication (Table A). Intracellular growth curves of the L. donovani field isolates and the reference lab strain. Using metacyclic promastigotes for infection, a decline in initial (24h) intracellular amastigote burden was observed for all the L. donovani strains tested. When ex vivo amastigotes (*) were used to infect host cells, a consistent increase in parasite burden was observed (Fig A). (DOCX) [file pone.0140139.s001.docx]

***Supplementary material***

**Comparative Fitness of a Parent *Leishmania donovani* Clinical Isolate and its Experimentally Derived Paromomycin-resistant Strain.**

S. Hendrickx^1^, A. Leemans ^1^, A. Mondelaers ^1^, S. Rijal ^2^, B. Khanal ^2^, J.C. Dujardin ^3,4^, P. Delputte  ^1^, P. Cos ^1^, L. Maes ^1*^

Monitoring the *in vitro* amastigote multiplication of a paromomycin (PMM)-susceptible and corresponding PMM-resistant *Leishmania donovani* strain in primary peritoneal mouse macrophages revealed a consistent decrease in amastigote burden over time. This observation required confirmation in a broader set of field isolates of *L. donovani* with different background of resistance for antimony (Sb), miltefosine (MIL) and PMM (Table S1). Being aware of the loss of virulence in promastigotes after long-term *in vitro* cultivation, *ex vivo* amastigotes were used for infection of those strains that had already been established in Balb/c mice or hamsters, *i.e.* our reference laboratory strain and the BPK275/0 Cl_18_ PMM-resistant strain that was originally selected *in vitro* after successive selection cycles on intracellular amastigote level. The other isolates were only available as axenic promastigotes and optimal metacyclic stages were used to infect macrophages adopting an infection level of 15 promastigotes/macrophage.

Intracellular growth curves are presented in Figure S2. Infection of macrophages with *ex vivo* amastigotes resulted in increasing intracellular parasite burdens over time. On the contrary, infection with metacyclic promastigotes consistently resulted in a decrease of initial (24h) intracellular parasite burdens. The latter observation strongly advocates the use of *ex vivo* amastigotes for experimental *in vitro* and *in vivo* infections, particularly in the frame of resistance and drug screening studies.

.

**S1 File. Evaluation of intracellular amastigote replication for a selection of *L. donovani* field isolates.** Drug-susceptibility profile of the selected *L. donovani* field isolates to evaluate intracellular amastigote replication **(Table A).** Intracellular growth curves of the *L. donovani* field isolates and the reference lab strain. Using metacyclic promastigotes for infection, a decline in initial (24h) intracellular amastigote burden was observed for all the *L. donovani* strains tested. When *ex vivo* amastigotes (*) were used to infect host cells, a consistent increase in parasite burden was observed **(Fig. A)**.

| **Strain** | **Sb^V^** | **Sb^III^** | **MIL** | **PMM** | **Country** | **Origin** |
| --- | --- | --- | --- | --- | --- | --- |
| BPK275/0 Cl_18_ PMM-R | R | R | S | R* | Nepal | experimentally selected against PMM |
| LdL82 (MHOM/ET/67/L82) | S | S | S | S | Ethiopia | reference lab strain |
| BPK282/0 Cl_4_ | S | S | S | R | Nepal | patient Sb-relapse |
| BPK500/0 | S | S | S | S | Nepal | patient Sb-failure |
| BPK513/0 | S | S | S | S | Nepal | patient Sb-failure |
| NEP098/6 | nd | nd | S | S | Nepal | MIL relapse isolate |
| NEP087/0 Cl_4_ | nd | nd | S | S | Nepal | MIL relapse isolate (clone) |

**S1 file. Table A.**

**

**

**S1 file. Figure A.**
